# Effect of the loading duration on the linear viscoelastic parameters of tropical wood: case of *Tectona grandis L.f* (Teak) and *Diospyros mespiliformis* (Ebony) of Benin Republic

**DOI:** 10.1186/2193-1801-3-74

**Published:** 2014-02-07

**Authors:** Agapi Kocouvi Houanou, Adolphe Dèfodji Tchéhouali, Amos Erick Foudjet

**Affiliations:** Laboratory of Energy and Applied Mechanics-Polytechnic School of Abomey-Calavi, University of Abomey-Calavi, Abomey-Calavi, Benin Republic; CRESA Forest – Wood, University of Dschang, Dschang, Cameroon

**Keywords:** Tropical wood, Bending creep, Loading duration, Dynamic modulus of elasticity, Dynamic modulus of viscosity

## Abstract

Judicious and regulated use of wood as a building material is better than that of many other conventional materials in terms of environmental issues of this century. The study of the behavior of wood requires a better understanding of the characteristics in different possible cases of loading including loads applied instantly, loads applied for a short time and loads applied for a long time.

The purpose of this study is to evaluate the influence of the loading duration on the linear viscoelastic parameters of tropical wood in creep test. Creep tests conducted on two species of tropical wood, *Tectona grandis L.f* and *Diospyros mespiliformis*, were carried out for a total loading duration of 15 hours by subjecting samples to bending test through with equal strain in all sections. After measuring the instantaneous deflection, the other measurements were carried out at regular time each 30 minutes.

Each recorded deflection was converted into longitudinal deformation and the data were analyzed by considering fourteen loading durations.

Using the least squares method, the dynamic modulus of elasticity and the modulus of dynamic viscosity were determined for each loading time. The results showed that the loading time has no influence on the modulus of dynamic viscosity. On the other hand, the dynamic modulus of elasticity decreases and tends towards zero. Good agreement between creep test data and dynamic modulus of elasticity was found using mathematical function in power. Suitably, the “power” function established between the elastic dynamic modulus and the loading duration can be used to extrapolate deformations values.

## Introduction

Wood used as a building material is a natural resource with multiple benefits, including quoting for example, the significant reduction of the negative environmental impacts generally registered when using other current building materials.

Its use in the field of civil engineering dates for a very long time and is encountered in the construction of large structures such as houses, bridges etc. Deconstruction waste generated by the demolition of the wooden structures, at the end of their life are rare easy manageable waste. Depending on the destination, the wooden structures may be solicited by short loading duration, average loading duration or long loading duration.

Deformations under these charges consist of instantaneous deformations and deferred deformations. Those under short loading duration are controllable and taken into account when designing classic works but the control of the failure mechanism resulting of deferred deformations requires knowledge of the viscoelastic parameters.

Several studies have been conducted to demonstrate the viscoelastic behavior of wood in order to establish its main properties. Thus, to characterize the deferred behavior of wood, most of authors use creep tests at the expense of relaxation tests, because of the consistent loading mode with the solicitation conditions encountered in current usages of wood and easy implementation of experimental devices for deflection measurements with a view to calculating the corresponding deformations.

The explanation of the linear range of wood viscoelastic behavior was also subject to several investigations. As reported by Montero (2010), studies conducted by Kingston and Clarke (1961), Nakai and Grossman (1983) and Mukudai (1983) showed that the wood viscoelasticity is linear for lower loadings at 40% of the ultimate tensile strength and the Boltzmann superposition principle is valid according to Nakai and Grossman (1983).

While studying the timber rheology, Foudjet (1986) also studied the linearity of its viscoelastic behavior from creep tests on some tropical species (Azobé, Tali, Sapelli and Movingui). The obtained results show that the deferred behavior of timber is viscoelastic linear for stresses less than or equal to 35% of the ultimate tensile strength. These measurements were obtained on iso-stress samples (cantilever) stabilized at 12% of moisture and submitted longitudinally at different levels of confining stress (respectively 25%, 30%, 35% and 42% of the ultimate tensile strength).

As for Randriambololona (2003), he reported in his paper that the linearity limit of viscoelastic behavior of wood depends on the type of stress and is situated at a stress between 10% and 20% of the ultimate tensile strength when the test is carried out in compression and between 20% and 30% of the ultimate tensile strength when it is the bending or tensile creep test.

Placet (2006) reported in his paper that the viscoelastic behavior of wood is strongly influenced by temperature and moisture in addition to the loading duration in respect of the polymeric nature of these constituents. Indeed, he found out that the behavior of a viscoelastic material to higher temperature for short loading times is equivalent to that of the same material at lower temperature, but for longer times. This is the principle of time-temperature equivalence or the principle of time-temperature superposition (Dlouhà 2009; Placet 2006).

In the paper of Montero (2010), it is said that when the water content is below the fiber saturation point, it influences the viscoelastic behavior at two levels: the kinetics’ evolution and mecanosorptive effect due to the viscosity of wood which depends on its water content, but is also very sensitive to the variation of the water content. Thus, the viscoelastic compliance is seven times higher for wet creep (moisture 22%), the same with a dry creep (moisture 0, 5%).

As for mecanosorptive fact, the first works were published in 1960 by Armstrong and Kingston highlighting the influence of the variation of moisture in wood in its deferred behavior. In bending tests, they compare the creep of wood samples kept at constant moisture with the one that can dry during the test. From these results, it appears that the creep on these samples is at least two times greater than the ones maintained at constant moisture. A year later, Armstrong and Christensen (1961) by detailing previous studies have indicated that this increase depends on the rate of sorption and not the moisture of the loaded sample (Montero 2010). These results on mecanosorption paved the way for other studies, including those of Randriambololona (2003) devoted to modeling the deferred behavior of wood in a variable environment.

The viscoelastic model is by far the most widely used for modeling the mechanical behavior of wood. In fact, the linear viscoelastic behavior is represented generally by constructing a model consisting of an assembly of springs and dashpots. It is therefore an analog and symbolic model represented by a combination of springs and dashpots in series and in parallel more or less complex (Foudjet 1986; Placet 2006; Dlouhà 2009). The works of Haque et al. (2000), devoted to the comparison of the relevance of these different models plus an empirical model based on the equation of Bailer-Norton empirical model, it was found that the Kelvin model seems to be the best suited to interpolate the experimental curves (Moutee 2006; Husson 2009). Thus, several authors adopt the Kelvin’s model, or more precisely the series connection of *n* Kelvin elements to restore the viscoelastic behavior of timber in a creep test. However, identifying problems quickly become insoluble because one needs to determine at least as many coefficients as elements introduced, which may be unworkable especially in practice just for the fact that these parameters highly depend on the moisture and temperature. In this context, Foudjet (1986) showed a rheological model with maximum two (2) Kelvin-Voigt elements connected in series which was widely enough to represent the own linear viscoelastic behavior of wood.

Other studies carried out on polymers linear viscoelastic behavior have showed that creep compliance J(t), is only a function of time and not a function of the magnitudes of stress and strain (Bower 2002; Brinson and Brinson 2008; Chanda and Roy 2009) and the deformation (strain) depend on the applied stress (Barners et al. 1993; Chanda and Roy 2009).

Considering the results obtained by these previous works, the studies published by Houanou et al. (2012) were devoted to the identification of linear viscoelastic paremeters of two tropical woods at a given moisture and constantly held, under a steady applied load during the entire test period.

As for Eurocode 5 (1995), wood is classified according to the mechanical strength criteria defined by the rules which ensure the reliability with regard to the use for which it is intended. Indeed, this standard defines a coefficient (k_mod_), amending the strength, taking into account the class of service of loading duration and moisture of the wood.

This coefficient (k_mod_) is used to determine the design value X_d_ of a property of the material.1

Where,

*X*_k_: characteristic value of material’s property

γ_m_: partial coefficient applying to the property.

The aim of this study is to determine the mechanism to be taken into account of the effect of the loading duration in the design of a wooden structure in the linear viscoelastic area and not simply in the elastic area as things were done until now.

More precisely, the aim of this work is to study the influence of loading duration on the linear viscoelastic parameters (dynamic modulus of elasticity and dynamic modulus of viscosity), wood moisture and applied load being maintained constant throughout the creep test. Otherwise, it will be to model the behavior of each parameter as a function of loading duration to extrapolate the values of creep or deformation. Finally, it will help to deduce the length of a bending creep test two (2) points after which a Kelvin-Voigt model reflects optimally the linear viscoelastic behavior of wood. The study will identify a model for predicting the mechanical lapse of an element of structure subjected to such stresses and consequently will develop a suitable method to extrapolate longitudinal strain values. Also, this works will permit to determine the species which has the best linear viscoelastic parameters.

To achieve these objectives, we used the parameter identification approach described in Houanou et al. (2012). This approach is applied by following the observation windows creep carefully chosen and appropriate mathematical functions to build the extrapolation models.

## Material and methods

**Figure 1:**
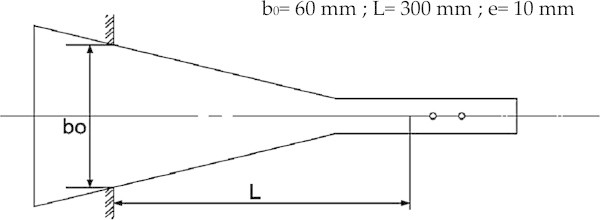
**Configuration of specimens.**

The samples were taken from the same board of the heartwood following the longitudinal direction (Houanou et al. 2012). For each species, twelve (12) experimental specimens were made up as shown in Figure 1.

The cut up samples provide the shape of an equally solicited beam in all its sections. The experimental specimens were dried to 12% moisture content in a modern dryer, in accordance with the normal conditions of temperature, pressure and speed drying. They were carefully surrounded by aluminum foil in order to maintain their water content under control.

**Figure 2:**
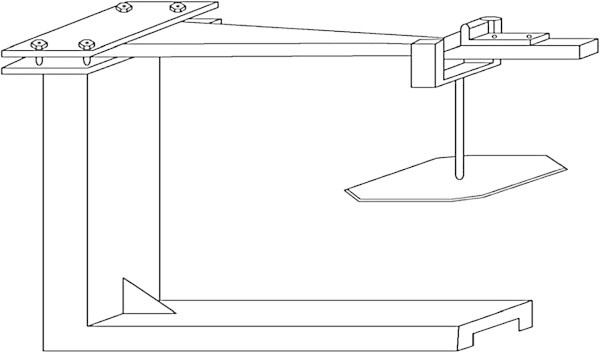
**Experimental device of bending creep test.**

The bending creep test consists on subjecting the sample to two points bending test. The samples are embedded at one end and 20% of the bending failure load (let 19.4 MPa for Teak and 25.2 MPa for Ebony) was applied at the other end. This load is applied to 300 mm from the other end of the beam (Figure 2) (Houanou et al. 2012).

Creep test were carried out within a total duration of 15 hours. The deflections were measured by means of a comparator with an accuracy of 1/100 mm every 30 minutes at mid-span of the beam after measuring the instantaneous deflection. Data have been treated by considering 14 periods: (0-2 h), (0-3 h), (0-4 h), (0-5 h), (0-6 h), (0-7 h), (0-8 h) (0-9 h), (0-10 h), (0-11 h), (0-12 h), (0-13 h), (0-14 h) and (0-15 h). Each period represents an observation window.

The specimens were weighed at the beginning and at the end of the creep test. The temperature is maintained at a constant value during manipulation. The deflections recorded have been converted to longitudinal deformation (Houanou et al. 2012) using the following formula:2

Where,

*σ*_*uc*_: ultime compressive stress (MPa)

*σ*_*ut*_: ultime tensile stress (MPa)

*f*: beam deflection (mm)

*L*: beam span (mm)

*h*: height of the beam (mm)

These arrows were used to calculate the viscoelastic creep compliance, *J*(*t*), using the following formula derived from Foudjet (1986):3

Where:

*ε* is the deformation calculated using (2),

*σ*_0_ means test loading.

Creep compliance *J*(*t*) is the sum of the instantaneous creep compliance *J*(*τ*) and the linear viscoelastic creep compliance *J*(*t>τ*) with τ, the time at which the instantaneous deformation is read (*τ*=15 *seconds*).

The mathematical expression of creep compliance is of the form (Foudjet 1986; Guitard 1987; Houanou et al. 2012):4

Equation 4 is derived from the rheological model of Zener. This model is the series combination of a spring characterizing the instantaneous deformation and Kelvin-Voigt model which represents the own creep of wood in the linear viscoelastic domain (Foudjet 1986; Guitard 1987; Houanou et al. 2012).

In this expression, “E_0_” is Hooke elasticity modulus; “E” means the dynamic elasticity modulus of the spring and “η” the dynamic viscosity modulus of the damper. For identification, we have:5

and6

For each observation window, the optimum values of the “E” and “η” of the expression of creep compliance in the linear viscoelastic field (6) are determined by adjusting the own creep compliance of using the method of least squares nonlinear as described in Houanou et al. (2012). The own creep compliance is calculated with (3) using the delayed deformation.

For a better leading of data analysis in purely linear viscoelastic field, a base change is made where the origin is at (0, 0) and the starting point of the experiment is now at (−τ; -j (τ)) in the new coordinate system. This change allows us to account for the period of reading the instantaneous deformation characterizing the purely elastic range.

The mathematical model to predict the evolution of the dynamic modulus of elasticity with the loading duration was established by means of the least squares method and the fitting equation deducted from the experimental curves can be expressed as follow (Polyanin and Manzhirov 2007):78

The iterations were conducted by considering the dynamic modulus of elasticity which coefficients of determination values are greater than 95%. The quality of each adjustment will be characterized by the coefficient of determination and the normality of residuals (Montgomery and Runger 2003). Then, using the coefficient of determination we will determine the optimal observations’ window.

In order to highlight the species, Teak or Ebony, which has better linear viscoelastic parameters, a comparative analysis of the curve of each of their linear viscoelastic compliance has been executed.

The tests were performed on two tropical species of Benin Republic, *Tectona grandis L.f* and *Disopyros mespiliformis*. Wood samples from the logyard ATC Wood Company. The *Tectona grandis L.f* is derived from plantations of Benin Wood National Office located in the central area of Benin Republic. However, *Diospyros mespiliformis* comes from natural forests in the northwest of Benin Republic. These logs were stored in the open air for one (1) to two (2) months before being boarded. Different boards are dried in the modern kiln of SECAL brand of ATC Wood Company.

## Results and discussion

The optimal values of “E” and “η” and the corresponding coefficient of determination indicating the reliability of simulated model are shown in Table 1.

**Table 1:** **Determined linear viscoelastic parameters “E” and “η”**

Loading duration (hours)		***Tectona grandis L.f***			***Diospyros mespiliformis***	
	E (MPa)	η (MPa.s)	Coefficient of determination R^2^	E (MPa)	η (MPa.s)	Coefficient of determination R^2^
**2**	8.23E+04	34.8E+07	96.00%	11.84E+04	39.48E+07	79.59%
**3**	7.49E+04	34.8E+07	96.30%	8.95E+04	39.48E+07	87.79%
**4**	6.92E+04	34.8E+07	97.31%	7.60E+04	39.48E+07	92.73%
**5**	6.56E+04	34.8E+07	98.42%	6.29E+04	39.48E+07	96.58%
**6**	6.30E+04	34.8E+07	99.01%	5.84E+04	39.48E+07	97.62%
**7**	6.16E+04	34.8E+07	99.31%	5.62E+04	39.48E+07	98.17%
**8**	6.06E+04	34.8E+07	99.45%	5.45E+04	39.48E+07	98.49%
**9**	5.96E+04	34.8E+07	99.53%	5.34E+04	39.48E+07	98.57%
**10**	5.88E+04	34.8E+07	99.55%	5.27E+04	39.48E+07	98.53%
**11**	5.78E+04	34.8E+07	99.39%	5.20E+04	39.48E+07	98.39%
**12**	5.71E+04	34.8E+07	99.10%	5.14E+04	39.48E+07	98.14%
**13**	5.65E+04	34.8E+07	98.76%	5.09E+04	39.48E+07	97.80%
**14**	5.60E+04	34.8E+07	98.35%	5.03E+04	39.48E+07	97.38%
**15**	5.56E+04	34.8E+07	98.08%	4.98E+04	39.48E+07	97.06%

We remark that the linear viscoelastic parameters (modulus of elasticity and dynamic viscosity) are different from one species to another for each given loading time. Thus, like other physical and mechanical properties of wood, the so-said parameters cannot escape from the variability among (Jacques 2003; Gardelle 2005; Almeida 2006; Laplanche 2006). This variability of the linear viscoelastic parameters of wood depends on the environment in which trees have evolved (soils, climates, Silvicultural practices…) but also on the part of the studied tree (trunk, roots, crown…) (Charron et al. 2003; Alteyrac 2005).

Also, this variability can be explained by the chemical composition of the studied species, especially the proportion of macromolecules constituents of the microstructure of their wood (Bower 2002; Brinson and Brinson 2008; Chanda and Roy 2009; Hang 2007; Chung 2010; Kumar and Gupta 2003; Lin 2011).

**Figure 3:**
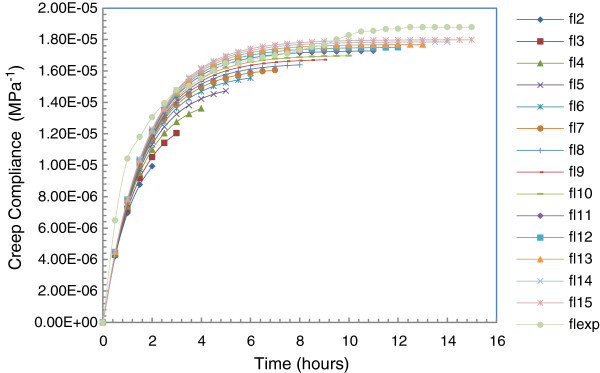
**Fitting curves of** ***Tectona grandis L.f*** **creep test in the linear viscoelastic phase.**

**Figure 4:**
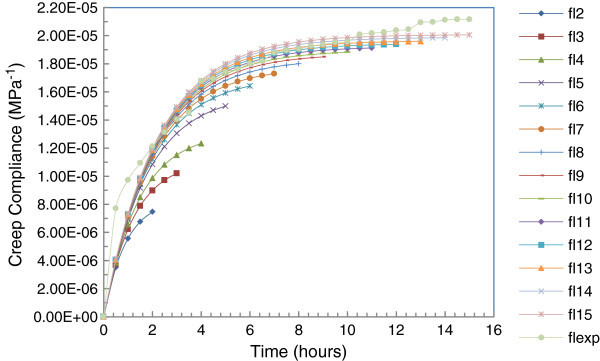
**Fitting curves of** ***Diospyros mespiliformis*** **creep test in the linear viscoelastic phase.**

Figures 3 and 4 show the curves of the different models and experimental values.

### Relevant assessment of the wood behavior in a bending creep test: optimal loading duration applicable in linear viscoelastic area

The characterization of differed mechanical behavior of wood goes through creep tests and relaxation. Unlike the rheological model and loading rate to use, the literature provides no information on the duration of each of these tests. This section will attempt to provide an indication of this determinant.

From Table 1, it is shown that the coefficient of determination (R^2^) of the linear viscoelastic models increases with the growth of the loading duration and reached a maximum value of 99.55% for a loading duration of 10 hours in case of *Tectona grandis L.f.* The maximum value is 98.57% for a loading duration of 9 hours in case of *Diospyros mespiliformis.* This variation of the coefficient of determination is due to the behavior of the various parameters of the linear viscoelastic of Kelvin-Voigt rheological model.

Thus, the results indicate that the analysis of the linear viscoelastic behavior of wood to characterize its own creep is effectively done by adopting the rheological model of Kelvin-Voigt provided that the loading time during the bending test two points is around 9 hours.

### The stability of the dynamic modulus of viscosity

From Table 1, the dynamic modulus of viscosity appears to be constant for all loading durations both of species *Tectona grandis L.f* and *Diospyros mespiliformis*. There are good agreement between these results and the fact that the viscosity of a material reflects the internal friction during the movement of atoms on a microscopic scale (Repellin 2006); the temperature is kept constant and nothing justifies a possible disruption of atoms’ constituents.

### Correlation between the loading duration and the dynamic modulus of elasticity

**Figure 5:**
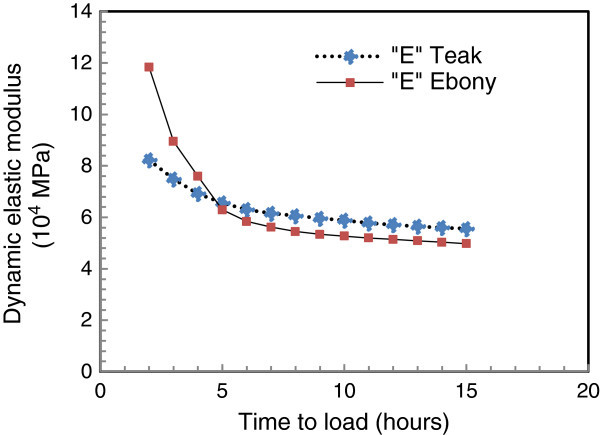
**Evolution of the modulus of elasticity dynamic with the loading duration.**

The dynamic modulus of elasticity decreases with the increase of loading duration in all cases of species as shown in Figure 5.

These results are consistent with those found at the end of Gardelle (2005) work who reported that the wood material is sensitive to charging time and under a given load, stiffness decreases over time resulting in further deformation. This loss of strength is accompanied by assuming an elastic behavior of wood, a decrease of stress in the material

These results are similar to those which were found in the case of the polymers whose mechanical properties depend not only on the loading duration but also on the temperature (Moutee 2006; Placet 2006; Montero 2010; Laplanche 2006).

The decrease of dynamic elasticity modulus is due to movements of the wood macromolecules’ constituents. These movements create the viscous behavior; one of the consequences is the sensitivity of mechanical properties to the application’s duration and speed of the load. For example, a longer period of application leads to a larger deformation therefore to a lower elasticity modulus (Dupeux 2008; Bower 2002; Sperling 2006).

### Modeling of the correlation between the loading duration and the dynamic modulus of elasticity

The values of the coefficients “a” and “b” of each function are indicated in Table 2.

**Table 2:** **Values of the coefficients of the models**

Model	Coefficient	***Tectona grandis L.f***	***Diospyros mespiliformis***
Power function *E*(*t*) = *at* ^*b*^	a	9.097E+04	8.302E+04
	b	−0.19	−0.194
Exponential function *E*(*t*) = *ae* ^*bt*^	a	7.7542E+04	6.5669E+04
	b	−0.026	−0.02

**Figure 6:**
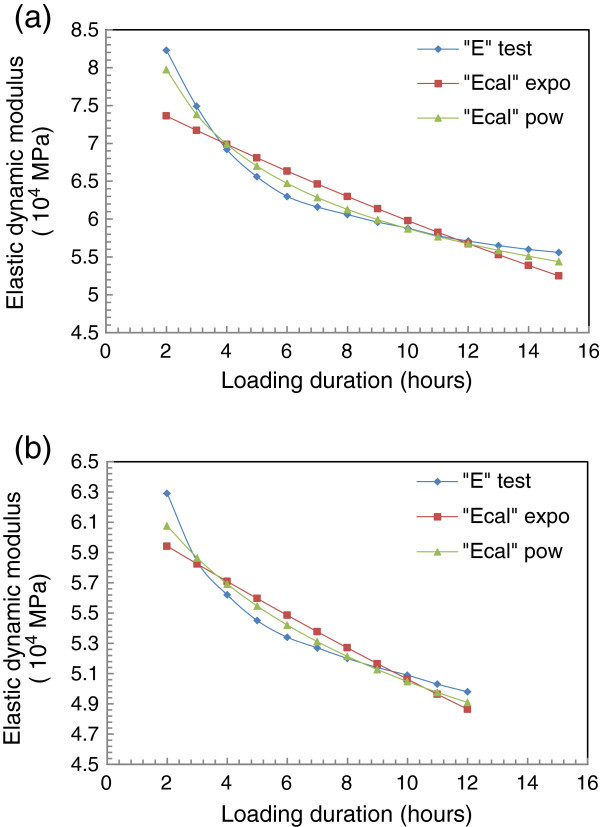
**Experimental and modeled curves of modulus of elasticity for dynamic with loading duration. (a)** Case of *Tectona grandis L.f*; **(b)** case of *Diospyros mespiliformis*

For each of the species studied, the Figure 6 presents experimental curves and modeled curves.

Calculated from equation 9 as follows:9

The different values of coefficient of determination for each model are shown in Table 3.

**Table 3:** **Coefficient of determination for the models**

Model	Coefficient of determination	
	***Tectona grandis L.f***	***Diospyros mespiliformis***
Power function	97.79%	95.31%
Exponential function	83.98%	87.59%

Then, the results show that at least 95% of the observations values are explained by the “power” model against 80% at least if we consider the “exponential” model no matter what species is carried out. From Tables 4 and 5 which provides the details of the calculations data indicate that 100% of the standardized residuals  belong to the [−2, + 2]. This proportion is significantly higher than the recommended value (95%) (Houanou et al. 2012). That confirmed that the residuals are normally distributed.

**Table 4:** **Calculations details of the adequacy of the model, case of power model**

***y***_***exp***_	Y_cal_	***ei*** = ***y***_***exp –***_ ***Y***_***cal***_			***d*** ^2^	***d***_***i***_
			**Case of** ***Tectona grandis L.f***			
8.23	7.97	0.2555	0.065	1.954	3.819	0.021
7.49	7.38	0.1068	0.011	1.214	1.474	0.009
6.92	6.99	−0.0705	0.005	0.644	0.415	−0.006
6.56	6.70	−0.1403	0.020	0.284	0.081	−0.011
6.3	6.47	−0.1722	0.030	0.024	0.001	−0.014
6.16	6.29	−0.1254	0.016	−0.116	0.013	−0.010
6.06	6.13	−0.0679	0.005	−0.216	0.047	−0.006
5.96	5.99	−0.0323	0.001	−0.316	0.100	−0.003
5.88	5.87	0.0065	0.000	−0.396	0.157	0.001
5.78	5.77	0.0119	0.000	−0.496	0.246	0.001
5.71	5.67	0.0365	0.001	−0.566	0.320	0.003
5.65	5.59	0.0621	0.004	−0.626	0.392	0.005
5.6	5.51	0.0902	0.008	−0.676	0.457	0.007
5.56	5.44	0.1220	0.015	−0.716	0.512	0.010
			**Case of** ***Diospyros mespiliformis***			
6.29	6.08	0.214	0.046	0.904	0.817	0.0228
5.84	5.86	−0.024	0.001	0.454	0.206	−0.0026
5.62	5.69	−0.072	0.005	0.234	0.055	−0.0076
5.45	5.55	−0.096	0.009	0.064	0.004	−0.0102
5.34	5.42	−0.081	0.007	−0.046	0.002	−0.0086
5.27	5.31	−0.041	0.002	−0.116	0.014	−0.0044
5.2	5.21	−0.014	0.000	−0.186	0.035	−0.0015
5.14	5.13	0.013	0.000	−0.246	0.061	0.0014
5.09	5.05	0.042	0.002	−0.296	0.088	0.0045
5.03	4.98	0.055	0.003	−0.356	0.127	0.0058
4.98	4.91	0.071	0.005	−0.406	0.165	0.0075

Where,

*y*_*exp*_: creep value obtained experimentally;

*Ycal*: creep value calculated using the model; sample means of observations;: standardized residual;: variance.

**Table 5:** **Calculations details of the adequacy of the model, case of exponential model**

***y***_***exp***_	Y_cal_	***ei*** = ***y***_***exp –***_ ***Y***_***cal***_			***d*** ^2^	***d***_***i***_
			**Case of** ***Tectona grandis L.f***			
8.23	7,36	0,8687	0,755	1,954	3,819	0,026
7.49	7,17	0,3176	0,101	1,214	1,474	0,009
6.92	6,99	−0,0683	0,005	0,644	0,415	−0,002
6.56	6,81	−0,2489	0,062	0,284	0,081	−0,007
6.3	6,63	−0,3342	0,112	0,024	0,001	−0,010
6.16	6,46	−0,3039	0,092	−0,116	0,013	−0,009
6.06	6,30	−0,2380	0,057	−0,216	0,047	−0,007
5.96	6,14	−0,1764	0,031	−0,316	0,100	−0,005
5.88	5,98	−0,0989	0,010	−0,396	0,157	−0,003
5.78	5,83	−0,0454	0,002	−0,496	0,246	−0,001
5.71	5,68	0,0341	0,001	−0,566	0,320	0,001
5.65	5,53	0,1197	0,014	−0,626	0,392	0,004
5.6	5,39	0,2117	0,045	−0,676	0,457	0,006
5.56	5,25	0,3100	0,096	−0,716	0,512	0,009
			**Case of** ***Diospyros mespiliformis***			
6.29	5,94	0,348	0,121	0,904	0,817	0,0265
5.84	5,82	0,016	0,000	0,454	0,206	0,0012
5.62	5,71	−0,089	0,008	0,234	0,055	−0,0068
5.45	5,60	−0,146	0,021	0,064	0,004	−0,0111
5.34	5,49	−0,145	0,021	−0,046	0,002	−0,0111
5.27	5,38	−0,107	0,011	−0,116	0,014	−0,0081
5.2	5,27	−0,070	0,005	−0,186	0,035	−0,0053
5.14	5,17	−0,026	0,001	−0,246	0,061	−0,0020
5.09	5,06	0,027	0,001	−0,296	0,088	0,0020
5.03	4,96	0,067	0,004	−0,356	0,127	0,0051
4.98	4,86	0,115	0,013	−0,406	0,165	0,0088

Where,

*y*_*exp*_: creep value obtained experimentally;

*Ycal*: creep value calculated using the model; sample means of observations;: standardized residual;: variance.

From these results, the “power” model is more appropriate than the “exponential” model although thermodynamically inappropriate, just because the power model type is a global smoothing which is more sensitive to uncertainties on the points than the law of the exponential type (Genevaux 1989). For the fact of its regularity giving it a greater ability to conduct longer term extrapolations, the power law or parabolic model should also fit very well in modeling of linear viscoelastic parameters (Schniewind and Barrett 1972; Genevaux 1989; Placet 2006). The values of the dynamic modulus of elasticity can be adjusted based on this model in order to find best extrapolated values when predicting the deformation of wooden structures in the linear viscoelastic area.

### Extension method validity: extrapolation of creep values

The performance analysis of extension models studied in this section shows the importance of using the global model of evolution of viscoelastic linear parameters based on the loading duration at the expense of models with constant parameters adjusted by timeslots.

The analysis of extension model performance is to show that the extended values are closer to experimental values than the other calculated values from models established according to the loading duration (for each observations window). To achieve this, we denote by:

*y*_*exp*_: creep value obtained experimentally;

*Y*_*cal*_, 2 h, *Y*_*cal*_, 9 h, *Y*_*cal*_, 10 h: creep value calculated using the model of 2 h, 9 h or 10 h;

*Y*_*cal*_, pow, *Y*_*cal*_, expo: creep value calculated using power or exponential models;

*e*: relative difference between the calculated and experimental values.The relative deviation is calculated using the following equation10

Table 6 below shows the various indicators of assessment in considering the models based on data collected with the loading time of 2 hours and 10 hours for *Tectona grandis L.f* and 2 hours and 9 hours for *Diospyros mespiliformis*. 10 hours and 9 hours represent respectively the loading duration which permit to have the optimal values of the linear viscoelastic parameters of *Tectona grandis L.f* and *Diospyros mespiliformis*.

**Table 6:** **Calculations of the implementation details**

***Duration (h)***	(a) ***Tectona grandis L.f***								
	***y***_***exp***_	Y_cal_, 2h	***e***	Y_cal_, 10h	***e***	Y_cal_, pow	***e***	Y_cal_, expo	***e***
1	1,04E-05	6,96E-06	33%	7,75E-06	26%	6,70E-06	36%	7,18E-06	31%
2	1,31E-05	9,94E-06	24%	1,20E-05	8%	1,01E-05	22%	1,06E-05	19%
3	1,48E-05	1,12E-05	24%	1,43E-05	4%	1,22E-05	18%	1,24E-05	16%
4	1,55E-05	1,17E-05	24%	1,55E-05	0%	1,35E-05	13%	1,35E-05	13%
5	1,65E-05	1,20E-05	27%	1,62E-05	2%	1,45E-05	12%	1,43E-05	14%
6	1,67E-05	1,21E-05	28%	1,66E-05	1%	1,52E-05	9%	1,48E-05	11%
7	1,70E-05	1,21E-05	29%	1,68E-05	1%	1,57E-05	7%	1,53E-05	10%
8	1,73E-05	1,21E-05	30%	1,69E-05	3%	1,62E-05	6%	1,58E-05	9%
9	1,76E-05	1,21E-05	31%	1,69E-05	4%	1,66E-05	6%	1,62E-05	8%
10	1,83E-05	1,21E-05	34%	1,70E-05	7%	1,70E-05	7%	1,67E-05	9%
11	1,85E-05	1,21E-05	34%	1,70E-05	8%	1,73E-05	7%	1,71E-05	8%
12	1,87E-05	1,22E-05	35%	1,70E-05	9%	1,76E-05	6%	1,76E-05	6%
13	1,88E-05	1,22E-05	35%	1,70E-05	9%	1,79E-05	5%	1,81E-05	4%
14	1,88E-05	1,22E-05	35%	1,70E-05	9%	1,81E-05	3%	1,86E-05	1%
15	1,88E-05	1,22E-05	35%	1,70E-05	9%	1,84E-05	2%	1,90E-05	−1%
***Duration (h)***	**(b)** ***Diospyros mespiliformis***								
	***y***_***exp***_	**Y**_**cal**_ **, 2h**	***e***	**Y**_**cal**_ **, 9h**	***e***	**Y**_**cal**_ **, pow**	***e***	**Y**_**cal**_ **, expo**	***e***
1	9,74E-06	5,58E-06	43%	7,22E-06	26%	6,40E-06	34%	6,90E-06	29%
2	1,21E-05	7,47E-06	38%	1,17E-05	4%	1,01E-05	17%	1,08E-05	11%
3	1,40E-05	8,11E-06	42%	1,44E-05	−2%	1,25E-05	11%	1,32E-05	6%
4	1,68E-05	8,33E-06	50%	1,61E-05	4%	1,42E-05	15%	1,47E-05	12%
5	1,74E-05	8,41E-06	52%	1,71E-05	2%	1,54E-05	11%	1,57E-05	10%
6	1,80E-05	8,43E-06	53%	1,77E-05	2%	1,64E-05	9%	1,65E-05	9%
7	1,88E-05	8,44E-06	55%	1,81E-05	4%	1,71E-05	9%	1,71E-05	9%
8	1,91E-05	8,44E-06	56%	1,83E-05	4%	1,77E-05	7%	1,76E-05	8%
9	1,94E-05	8,45E-06	56%	1,85E-05	4%	1,82E-05	6%	1,80E-05	7%
10	1,96E-05	8,45E-06	57%	1,86E-05	5%	1,87E-05	5%	1,85E-05	6%
11	2,02E-05	8,45E-06	58%	1,86E-05	8%	1,91E-05	5%	1,89E-05	6%
12	2,04E-05	8,45E-06	59%	1,87E-05	8%	1,94E-05	5%	1,93E-05	5%
13	2,09E-05	8,45E-06	60%	1,87E-05	11%	1,98E-05	6%	1,97E-05	6%
14	2,11E-05	8,45E-06	60%	1,87E-05	11%	2,01E-05	5%	2,01E-05	5%
15	1,88E-05	8,45E-06	55%	1,87E-05	0%	2,03E-05	−8%	2,05E-05	−9%

From Table 6, we remark that the extended values calculated through power model and exponential one are closer to the experimental values than those values calculated directly from the model of 2 hours or 10 hours, for all the species studied.

**Figure 7:**
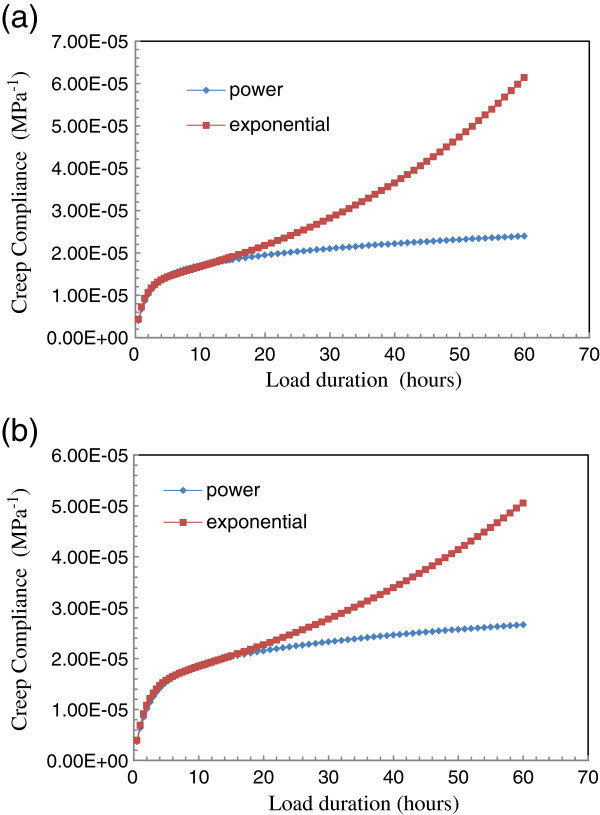
**Extension creep curves for power model versus exponential one. (a)** Teak **(b)** Ebony.

Figure 7 permits to examine the predictive ability of extension functions based on the power or exponential model for long loading times. It shows that the extension curve based on the exponential model changes concavity after 4 hours and 6 hours and half respectively for *Tectona grandis* L.f and *Diospyros mespiliformis*. For each species studied, this new concavity is turned upwards instead of the right, the correct direction of the creep curve concavity. However, the curve of extension function based on power model retains its shape with the concavity turns towards right.

Thus, the value of E will be updated using the power model, *E*(*t*) = *at*^*b*^, to extrapolate creep value. This updated value of E is brought in the strain expression of course that the viscosity is constant.

### Comparative analysis of Teak and Ebony creep

**Figure 8:**
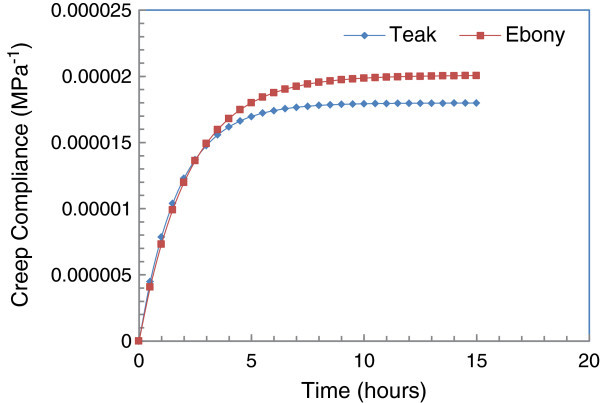
**Creep compliance curve of Teak versus Ebony.**

Analysis of Eq. 4, it appears that the linear viscoelastic compliance *J(t)* and the deformation *ε(t)* move in the same direction. In other words, greater is the deformation when the linear viscoelastic compliance is higher and vice versa. Thus, a kind of linear viscoelastic compliance *J*_*1*_*(t)* has better linear viscoelastic parameters compared to another linear viscoelastic compliance *J*_*2*_*(t)* where *J*_*1*_*(t)* is less than *J*_*2*_*(t).* According to Figure 8, Ebony has slightly better viscoelastic parameters than those of Teak for some loading times inferior to 2 h 42 mn. When the loading times are superior to 2 h 42 mn, the Teak has clearly the better linear viscoelastic parameters than those of Ebony.

## Conclusion

This study has emphasized the influence of the loading duration on the linear viscoelastic parameters of wood. Wood samples suitably cut up have been subjected to bending creep test. The investigations have been carried out on two tropical wood species (*Tectona grandis* L.f and *Diospyros mespiliformis*). Mathematical model to predict the evolution of the dynamic modulus of elasticity with the loading duration was established by means of the least squares method.

The results showed that the dynamic modulus of viscosity keeps a constant value when the loading durations increase and that, for the creep test period superior to 1 hour. However, the dynamic modulus of elasticity decreases according to the power function *E*(*t*) = *at*^*b*^ where *a* > 0;*b* < 0 *and t* > 0 the exponential function *E*(*t*) = *ae*^*bt*^ with *a*>0;*b* < 0 *and t* ≥ 0. Each of these laws creates a package that tends to zero when the loading time is relatively long. However, the power law is more appropriate. Further, the results showed that one can characterize the free creep of wood trough the assessment of the linear viscoelastic behavior adopting the rheological model of Kelvin-Voigt in bending creep test when it is carried out within a maximum loading duration of 9 hours.

Finally, studies have shown that for some short loading times inferior to 2 h 42 mn, the linear viscoelastic parameters of Ebony are slightly better than those of Teak. But beyond that, the linear viscoelastic parameters of Teak are frankly better than those of Ebony.
